# The Validity of the WHO-5 as an Early Screening for Apathy in an Elderly Population

**DOI:** 10.1155/2012/171857

**Published:** 2012-09-10

**Authors:** Ramona Lucas-Carrasco, Peter Allerup, Per Bech

**Affiliations:** ^1^Department of Methodology and Behavioral Sciences, University of Barcelona, SGR 822 Generalitat de Catalunya, 08035 Barcelona, Spain; ^2^Department of Education, Aarhus University, Campus Emdrup, 2400 København, NV, Denmark; ^3^University of Copenhagen, Psychiatric Research Unit, Mental Health Centre North Zealand, Dyrehavevej 48, 3400 Hillerød, Denmark

## Abstract

*Aim*. The objective of our study has been to evaluate the WHO-5 as a new early screening instrument for apathy in a group of elderly persons. *Methods*. The WHO-5 was compared to the Geriatric Depression Scale (GDS-15). The GDS contains five items measuring well-being and ten items measuring depression. The internal validity of the WHO-5 (total score being a sufficient statistic) was evaluated with both parametric and nonparametric item response theory models. The external validity of the WHO-5 and the GDS was evaluated by ROC using depression as index of validity. *Results*. The item response theory analyses confirmed that the total score of the WHO-5 is a sufficient statistic. The ROC analysis shows an adequate sensitivity (61%) and specificity (84%). The GDS_15_ and its two subscales obtained low sensitivity (25–42%), but high specificity (90–98%). *Conclusion*. The WHO-5 was found both internally and externally valid when considering decreased positive well-being to be an early indication of apathy reflecting that the wind has begun to be taken out of the “motivation sail.”

## 1. Introduction

Cognitive disorders for example, dementia, stroke, Parkinson's Disease or epilepsy are often accompanied by noncognitive syndromes such as depression and apathy. Measures of depression severity or severity of apathy have been found useful by their differentiating between the overlapping non-cognitive symptoms and the cognitive symptoms in the clinical management of dementia, stroke, Parkinson's Disease, or epilepsy.

Both depression and apathy are components of abulia, a term used by neurologists and neuropsychiatrists to denote lack of spontaneous goal-directed behaviour [1, 2].

The Geriatric Depression Scale (GDS) was developed by Yesavage et al. [3] and has been used in many clinical trials aimed at identifying depression in patients with cognitive disorders, especially dementia. Weeks et al. [4] reduced the original 30 item GDS to a 15 item version (GDS-15). The GDS-15 covers two subscales, namely, 10 items measuring specific depression symptoms and 5 items measuring psychological well-being.

The syndrome of apathy was measured [5] by the Apathy Evaluation Scale (AES). This scale is still the only specific apathy scale. The AES contains 18 items. Three of these items are negatively formulated, such as lack of putting effort into anything (anergy). The remaining 15 items are all positively formulated. Eight of these items are concerned with being interested in things and 7 items cover initiative, motivation, or emotional contact.

The term clinimetrics was introduced by Feinstein [6, 7], with focus on the clinical markers in clinical medicine before more or less sophisticated psychometric models were applied. In clinical psychometrics [8] a constructive dialogue is introduced in an attempt to develop the best possible instruments for the measurement of such syndromes as depression or apathy. The item response theory model [8] is an analysis of how to add symptoms to make a total score. Within this model, items with local dependency should be reduced. Clinically we are dealing with local dependency as a measure (correlation) of to what extent the score on one item can automatically predict the score on another item. Many of the items in AES have a clear local dependency, reflected by the very high alpha coefficients obtained (from 0.86 to 0.94) by Marin et al. [5]. It is always possible to achieve very high alpha coefficients by simply using questions which are merely variants of a simple, too restricted area [9]. In contrast, item response theory models require local independency implying that each item provides new information about the dimension being extensively examined [8].

We have from a clinical point of view considered apathy to be the negative formulation of psychological general well-being, that is, apathy is regarded as passive pessimism. Hall et al. [10] have recently evaluated the clinical validity of widely used well-being scales and identified the five items in the WHO-5 scales as having the highest content validity of psychological well-being when compared to 21 other scales with a much larger number of items such as the 36-item Medical Outcomes Study (SF-36) or the World Health Organization Quality of Life Scale (WHOQoL). We have focused on the WHO-5 as an indicator of apathy. 

The objective of our study is to evaluate the WHO-5 as a new early screening tool for apathy in a group of elderly persons. It is hypothesized that the sensitivity of the WHO-5 is higher than the sensitivity of GDS-15 but that the GDS-15 would have a higher specificity than the WHO-5 in a group of elderly persons.

## 2. Materials and Methods

### 2.1. Study Population

Participants were recruited from community centres and primary care centres in Spain. At each recruitment site, participants were invited to take part in the study by a staff member, who explained the purpose of the study. Participants were included if they were 65 years of age or older, able to read and write, and willing to provide written informed consent. Participants were excluded if the primary care physicians found that they had a severe cognitive impairment and/or serious auditory or visual impairment. Thus, participants with neurological diseases (e.g., Parkinson's Disease or epilepsy) but without severe cognitive impairment were also included.

### 2.2. Measures

#### 2.2.1. The WHO-5 Well-Being Questionnaire [8, 11]

A self-administered five-item scale; each item assesses the degree of positive well-being during the past 2 weeks on a six-point Likert scale graded from 0 (at no time) to 5 (all of the time); the raw score ranges from 0 to 25 of well-being. However, in order to obtain a score on a scale from 0 (worst thinkable well-being) to 100 (best thinkable well-being) these raw scores have been multiplied by 4.

#### 2.2.2. Geriatric Depression Scale (GDS-15) [3]

A 15-item questionnaire that measures depressive symptoms; answers are reported on a yes/no scale with high scores indicating more severe depression because the 5 items dealing with positive well-being have to be reversed for the total GDS-15 score. The time frame for the measure is the present (i.e., the past few days). A cut-off score of 5 was used to identify a sample of nondepressed (GDS-15 < 5) versus depressed (GDS-15 ≥ 5) participants. The Spanish version, validated among elderly persons from primary care centres, was used [12]. We have also focused on the 10 items for depression and the 5 items for well-being separately.

#### 2.2.3. Sociodemographic Information and Information about Subjective Perception of Health

The participants reported whether or not they felt healthy or unhealthy, answering the question: *In general, do you consider yourself to be currently healthy or unhealthy?* Chronic conditions such as hypertension; arthritis; diabetes; depression; cancer; heart, lung, gastric, thyroid, and kidney diseases as well as neurological disease (e.g., Parkinson's Disease or epilepsy) and hearing and vision problems were self-reported (“yes/no”). 

Participants completed measures in small groups at each participating centre. One researcher was present at each session in case participants requested any assistance. All measures were self-reported. All participants provided written informed consent. 

For standardization of the WHO-5 we used the WHOQOL item of general quality of life all things considered.

“Over the past two weeks how would you rate your quality of life?” 1 = very poor, 2 = poor, 3 = neither poor nor good, 4 = good, 5 = very good.

### 2.3. Statistical Analyses

#### 2.3.1. Item Response Theory Models

One of the basic principles behind the one-parameter Rasch model [13] and the nonparameter Mokken model [14] is that items with low prevalence have to be proceeded by scores on high prevalence items in every subgroup of patients [15]. This structure (Guttman structure [16]) is undertaken in terms of tests for rankings under the Mokken model and as a full parametric test in the Rasch model [15, 17–19].

#### 2.3.2. The One-Parameter Rasch Model

The Rasch analysis was carried out by analysing pairwise item comparisons [18–20]. Using this method, the model fit was evaluated through numerical test statistics and, graphically through analysis of the Item Characteristic Curves (ICC). During this process each item was inspected for different item discriminations (i.e., different slopes of the ICC curves). Evaluation of item bias with respect to gender was evaluated by comparing ICC curves from male and females. On successful acceptance of these two tests the WHO-5 was considered unidimensional [8, 20]. 

#### 2.3.3. The Nonparametric Mokken Model

The test of unidimensionality according to the Mokken model is carried out by the Loevinger coefficient of homogeneity which is basically a correlation analysis derived from the cumulative scaling [14]. We have used the Mokken scale analysis for polytomous items (MSP), version 3.0 [21]. According to Mokken, a coefficient of homogeneity between 0.30 and 0.39 is only just acceptable, a coefficient of homogeneity between 0.40 and 0.49 is acceptable, and a coefficient of homogeneity of 0.50 or higher is excellent [14]. In contrast to the Rasch analysis the Mokken model has no testability approach for factors outside the interval data set, for example, the impact of gender.

The external validity of the WHO-5 and the GDS was evaluated by a ROC (Receiver Operating Characteristic) curve.

## 3. Results

### 3.1. Sample Characteristics

The sample consisted of 191 elderly participants, 61.8% were female. Mean age for the entire sample was 74.6 years (standard deviation ±7.1; range of 65–95), with no significant differences in age between males and females (73.8 versus 75.1; *t* = −1.191, df 189, *P* = 0.235, two-tailed). Fifty one percent of participants were married. Sixty-six percent considered themselves to be *healthy*, but 95.3% reported having one or more of the chronic health conditions on the comorbid list, namely, arthritis 57.6%; hypertension 47.1%; eye problems 41.9% and hearing problems; 23.6% heart problems 20.9%; and depression 18.8%. On the GDS-15, 22.5% had significant depressive symptoms (GDS ≥ 5). On the WHO-5, 24.6% scored below 50 (Table 1).

In the Mokken analysis the mean scores have the same rankings of these two items (Table 2(a)). Apart from this, the rankings in Tables 2(a) and 2(b) are similar. The coefficient of homogeneity is 0.59 for all 5 items in the WHO-5 and, as indicated in Table 2(a), the coefficients for the individual items are all higher than 0.50, that is, an acceptable unidimensionality. For the Rasch analysis the WHO-5 also fulfilled the criterion of unidimensionality (*P* > 0.05) and no gender bias was seen.

### 3.2. ROC Results

Table 3 shows the ROC analysis for the calculation of sensitivity and specificity. The WHO-5 obtained both adequate sensitivity and specificity for the cut-off score of ≤50. Thus when using the patients' own self-reported depression scores as an index of validity, the sensitivity was 61% and the specificity was 84% for WHO-5. Using the self-reported depression scores, the GDS-15 obtained a high specificity but a very low sensitivity. This pattern was also obtained for the GDS-10 (depression subscale) and the GDS-5 (well-being subscale), as indicated in Table 3.

Finally we found that the mean score on WHO-5 for males (*N* = 73) was 65.7 (20.8) and for females (*N* = 118) 60.2 (20.4). This difference was close to be statistically significant, *P* = 0.07.

Our results with the item response theory model (Rasch) indicate that this difference was not due to item bias within gender.

### 3.3. Standardization and Validation

Using the WHOQOL item of general quality of life as an external index of validation, we found that the number of observations within the WHOQOL BREF item of general quality of life was too small as regards category 1 = very poor and category 5 = very good. In the category 2 = poor quality of life (*N* = 13), the WHO-5 mean score was 37.5 (21.4), for category 3 = neither good nor poor (*N* = 93), the WHO-5 was 59.6 (20.8), and for category 4 = good quality of life (*N* = 72) the WHO-5 was 68.9 (16.2). The difference between these three answer categories on the WHO-5 is statistically significant (*P* < 0.001).

## 4. Discussion

Both the WHO-5 and the GDS-15 had a high degree of applicability in the group of elderly persons investigated in this study. The limitation of using such self-reported questionnaires is obviously patients with severe cognitive impairments. In their study on the association between apathy and depression, Marin et al. [22] used the Hamilton Depression Scale, that is, a clinician administered scale. However, in both the Hamilton Depression Scale as well as the Montgomery-Åsberg Depression Scale [23] many items are actually self-reported symptoms.

Both the WHO-5 and the GDS-15 questionnaires are patient friendly for administration. Thus, the WHO-5 only contains 5 items, but with multicategory responses, whereas the GDS-15 contains items with dichotomized responses. In the case of the more complicated Beck Depression Inventory (BDI), the authors recommended [24] that a staff member should read out the questions to the depressed patients. If necessary this approach, which is possible for the AES, might also be used for the WHO-5 or the GDS-15.

In their study evaluating the symptom overlap between apathy and depression in a correlation analysis between the Apathy Evaluation Scale (AES) and the Hamilton Depression Scale (HAM-D), Marin et al. [22] identified the HAM-D items of work and interests, psychomotor retardation, and lack of energy with significant overlap to the AES total score. The other core items of depression in the HAM-D, namely, depressed mood, guilt feelings, and psychic anxiety had less overlap with the total score on AES [22]. 

The concept of apathy seems to imply that the passive pessimism, or lack of motivation, is not treatable. In their treatment approach to patients with apathy. Marin et al. [25] correctly state that apathy and abulia are placed on dimensions of severity with abulia considered as an indicator of severity [2]. Thus, abulia was considered by Eliot to be a noncognitive state because it is characterized by an impairment of mood and will [26]. In cases of “senile depression” or apathy, the stimulating antidepressants such as bupropion and monoamino-oxidase inhibitors are preferable, as shown by Marin et al. [25].

As discussed by Schneider et al. [27], the WHO-5 well-being scale is a most valid instrument as a first screening test in patients with Parkinson's disease where a more depression specific questionnaire such as the Beck Depression Inventory [24] has too low sensitivity, probably because of its length (21 items) and complexity [27]. We have previously found the 10-item Major Depression Inventory superior to the much longer Zung Depression Scale in patients with Parkinson's disease [28].

The present study on elderly persons without severe cognitive symptoms has found the WHO-5 to be applicable as observed by Schneider et al. [27]. We have found a sensitivity and specificity for depression of 61% and 84%, respectively, as adequate comparable to the results by Schneider et al. [27].

Compared to the Beck Depression Inventory or the Zung Depression Scale, the Geriatric Depression Scale is much more applicable in a population of elderly persons such as the group tested in this study. The 15-item GDS was found as applicable as the WHO-5. However, the very low sensitivity of the GDS-15 and the GDS-10 as well as the GDS-5 might indicate that these checklist versions are not to be used as the very first screening instrument for subjective apathy. On the other hand, the very high specificity of the GDS does indicate that the scale should be considered as the next scale in a stepped approach with more and more specific instruments.

## 5. Conclusion

In conclusion, we have shown that the WHO-5 fulfilled the item response theory model in the elderly with an invariant item ordering in agreement with the subjective aspect of the dimension of apathy. As a very short scale, the WHO-5 was found recommendable as the very first screening scale, indicating whether the wind has begun to be taken out of the “motivation sail.” Because apathy has so great an overlap with depression and because antidepressants might be considered in such cases, the Geriatric Depression Scale, as found in our study, should be considered as the next step in the diagnostic process. 

## Figures and Tables

**Table 1:** Participant characteristics: sociodemographic and health status variables.

	Total sample, *n* = 191
Age: mean (SD)	74.6 (7.1)
Range	65–95
Gender, *n*(%)	
Male	73 (38.2)
Female	118 (61.8)
Marital status, *n*(%)	
Single	18 (9.4)
Married	98 (51.3)
Partnered (other than married)	2 (1.0)
Separated/divorced	5 (2.6)
Widowed	68 (35.6)
Education, *n*(%)	
Primary school	105 (55.0)
Secondary school and higher	39 (20.4)
Less than primary school	47 (24.6)
Self-perceived health, *n*(%)	
Healthy	126 (66.0)
Unhealthy	65 (34.0)
Self-reported depression (%)	18.8
GDS-15 ≥ 5 (%)	22.5
WHO-5 ≤ 50 (%)	24.6

**(a):** 

	Item 1 good spirits	Item 5 interested in things	Item 2 relaxed	Item 3 energy	Item 4 fresh and rested
	3.17 (1)	3.17 (1)	3.15 (3)	3.06 (4)	3.02 (5)
Coefficients of homogeneity	0.63	0.63	0.62	0.55	0.53

**(b):** 

	Item 1 good spirits	Item 5 interested in things	Item 2 relaxed	Item 3 energy	Item 4 fresh and rested
	−0.25 (1)	−0.09 (2)	−0.07 (3)	0.09 (4)	0.32 (5)

**Table 3:** The ROC analysis concerning sensitivity and specificity of WHO-5 and GDS-15 with corresponding AUC (area under curves).

Scales	Sensitivity	Specificity	AUC
WHO-5 ≤ 50			
Using self-reported depression	0.61	0.84	0.73
Using GDS ≥ 5	0.82	0.83	0.88
GDS 15 ≥ 5			
Using self-reported depression	0.25	0.98	0.75
GDS 10 ≥ 5	0.25	0.98	0.75
GDS 5 ≥ 3	0.42	0.90	0.69
